# Maneuvering the Migration and Differentiation of Stem Cells with Electrospun Nanofibers

**DOI:** 10.1002/advs.202000735

**Published:** 2020-06-09

**Authors:** Jiajia Xue, Dario Pisignano, Younan Xia

**Affiliations:** ^1^ The Wallace H. Coulter Department of Biomedical Engineering Georgia Institute of Technology and Emory University Atlanta GA 30332 USA; ^2^ Dipartimento di Fisica Università di Pisa Largo B. Pontecorvo 3 Pisa I‐56127 Italy; ^3^ NEST Istituto Nanoscienze‐CNR Piazza S. Silvestro 12 Pisa I‐56127 Italy; ^4^ School of Chemistry and Biochemistry School of Chemical and Biomolecular Engineering Georgia Institute of Technology Atlanta GA 30332 USA

**Keywords:** cell differentiation, cell migration, electrospun nanofibers, scaffolding materials, stem cells

## Abstract

Electrospun nanofibers have been extensively explored as a class of scaffolding materials for tissue regeneration, because of their unique capability to mimic some features and functions of the extracellular matrix, including the fibrous morphology and mechanical properties, and to a certain extent the chemical/biological cues. This work reviews recent progress in applying electrospun nanofibers to direct the migration of stem cells and control their differentiation into specific phenotypes. First, the physicochemical properties that make electrospun nanofibers well‐suited as a supporting material to expand stem cells by controlling their migration and differentiation are introduced. Then various systems are analyzed in conjunction with mesenchymal, neuronal, and embryonic stem cells, as well as induced pluripotent stem cells. Finally, some perspectives on the challenges and future opportunities in combining electrospun nanofibers with stem cells are offered to address clinical issues.

## Graphical abstract

Enabled by their unique capability to mimic some features of the extracellular matrix, electrospun nanofibers have found widespread use as scaffolding materials for tissue regeneration. Here, a critical assessment of recent developments in using electrospun nanofibers to maneuver the migration and differentiation of various types of stem cells is offered.

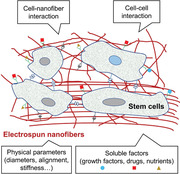

## 1 Introduction

Tissue regeneration can be considered as a medical procedure that integrates the principles of materials science, biology, and engineering to provide a roadmap for recovering the functions of damaged tissues and even organs.^[^ ^1^ ^]^ Key to the success of such a treatment is to understand the interactions between cells and their extracellular matrix (ECM) and how to recapitulate the essential features in manmade systems.^[^ ^2^ ^]^ Secreted by cells, ECM is a 3D fibrous network assembled from various types of proteins and proteoglycans. It not only serves as a structural support to the cells but also provides cues to regulate the attachment, spreading, migration, and differentiation of cells. As a critical requirement for tissue regeneration, the scaffolding materials must be made with the capability to mimic the ECM in regulating cellular behaviors by providing an appropriate microenvironment.

Many strategies for tissue regeneration are based on the culture of autologous cells in manmade scaffolds. A notable example can be found in BioSeed‐C, an engineered graft that contains autologous chondrocytes embedded in a bioresorbable scaffold based on polymer gel, and it has been applied to the treatment of degenerative and traumatic articular cartilage defects.^[^ ^3^ ^]^ In this case, an invasive procedure is typically required to collect autologous cells from the specific tissue, and sometimes a sufficient number of cells cannot be obtained for in vitro expansion or in vivo transplantation. As an alternative, stem cells show great potential as a viable source of cells for tissue regeneration because they can be conveniently harvested and expanded. In different variants, including adult, embryonic, and induced pluripotent, stem cells are known for their variations in capability to maintain their pluripotency through self‐renewal and to transform into specific phenotypes through differentiation.^[^ ^4^ ^]^ The behaviors of stem cells are largely determined by the local tissue microenvironments known as “stem cell niches.”

For the in vitro approach, stem cells are differentiated into the typical type(s) of cells in the scaffold outside the body and then the cellular construct will be transplanted into the body to facilitate the regeneration or repair process. In the case of an in vivo approach, the scaffold is implanted into the body to direct the migration of endogenous stem cells from their native niche toward the target site and then activate their function of proliferation and/or directional differentiation into the specific cell type(s).^[^ ^5^ ^]^ In addition to their role as the building blocks that directly participate in the regeneration process, stem cells can also work as a biological vehicle to provide stimulating signals to the target tissue in a paracrine fashion.^[^ ^5^ ^]^ To this end, it is of great importance to develop scaffolds capable of mimicking the native niches as close as possible for maneuvering the behaviors of stem cells, especially for directing their migration and controlling their differentiation.

Electrospun nanofibers are highly effective in this respect because of their unique ability to mimic the native ECM by offering a fibrous structure, a mechanical support, and, to a certain extent, chemical/biological cues. Specifically, electrospinning has emerged as a simple and versatile technique for the production of thin fibers with diameters ranging from tens of nanometers to a few micrometers.^[^ ^6^ ^]^ A wide variety of natural and synthetic polymers can be directly electrospun into nanofibers, making it possible to mimic the composition of the native ECM. In addition, the glycoproteins and other molecules existing in ECM, such as laminin and fibronectin, can be easily deposited onto the fiber surface or incorporated into the nanofibers. From the viewpoint of engineering, the physical parameters of a scaffold based on electrospun nanofibers, including the bulk composition, morphology, surface functionality, dimensionality, and mechanical strength can all be tailored to suit the target tissue. For example, the diameter of electrospun nanofibers can be readily controlled in the range of 50–500 nm to match the size of collagen fibers found in the native ECM. In addition, electrospun nanofibers can be easily collected as ordered arrays to mimic the orientations of ECM in various types of tissues. The ECM and ECM‐incorporated growth factors, together with cytokines, provide a number of functional cues that affect stem cell behavior. The surface chemistry of the electrospun nanofibers can also be tailored by functionalization through physical or chemical methods, endowing the surface with specific domains, such as ECM components, growth factors, DNA, and organic modifiers, to interact with or be recognized by stem cells. Furthermore, the high porosity and spatial interconnectivity make scaffolds of electrospun nanofibers a favorable microenvironment for cellular development. Not only limited to 2D mats, scaffolds with 3D architectures can also be fabricated from electrospun nanofibers to facilitate cell infiltration in the bulk. However, it should be pointed out that it is difficult for electrospun nanofibers to precisely mimic the tissue hierarchy in terms of multiple dimensions and structures. Nonwoven nanofiber sheets produced by electrospinning are usually below 1 mm in thickness, which is quite different from the native ECM in some tissues and inner organs. Despite the development of fibrous scaffolds with 3D architecture, they are still far away from matching the complexity of the real ECM. Although with this limitation, when combined with stem cells and growth factors, constructs based on electrospun nanofibers have been applied to promote the repair/regeneration of various types of tissues.

This article focuses on the use of electrospun nanofibers in controlling the migration and differentiation of stem cells. We start with a brief introduction to the physico‐chemical properties of electrospun nanofibers that are of critical importance to the proposed applications. Relevant issues include their biocompatibility, biodegradation kinetics, and mechanical properties, as well as their organization into controllable 2D and 3D architectures. We then discuss the mechanisms of cell migration and how to control cell migration by engineering the properties of electrospun nanofibers, as well as the highlight of a few specific applications involving stem cells. In Section 4, we discuss the use of electrospun nanofibers in controlling the differentiation of mesenchymal, neural, and embryonic stem cells, as well as induced pluripotent stem cells (iPSCs). At the end, we discuss the current challenges and issues that still need to be addressed in the design of scaffolds based on electrospun nanofibers and provide perspectives for future development, with an aim to maximize the potential of electrospun nanofibers and stem cells in clinical applications.

## 2 Electrospun Nanofibers as Scaffolding Materials for Stem Cells

Electrospinning typically involves the use of a high‐voltage power supplier, a syringe pump, a spinneret, and a conductive collector onto which the nanofibers are deposited.^[^ ^6^, ^7^ ^]^ When a polymer solution is pumped out through the spinneret, it tends to form a pendant droplet owning to the effect of surface tension. In the presence of a high voltage bias between the spinneret and the collector, charges of the same sign will be accumulated on the surface of the droplet. The charges repulse to each other and when the repulsion is strong enough to overcome the surface tension, the droplet will be deformed into a Taylor cone, and then a jet will be emanated from the apex of the cone. As the jet moves toward the collector, it will continuously decrease in diameter as a result of stretching caused by electrostatic repulsion, whipping, and solvent evaporation. Fibers with uniform diameters ranging from tens of nanometers up to a few micrometers can be readily produced by controlling the spinning parameters, including the concentration and flow rate of the polymer solution, the applied voltage, and the distance between the spinneret and the collector, among others.

Electrospun nanofibers have been successfully produced from a wide variety of materials, including various types of polymers, small molecules, ceramics, and composites.^[^ ^6^, ^7^ ^]^ This broad spectrum of materials is instrumental to the use of electrospun nanofibers as scaffolding constructs for stem cells in the context of tissue regeneration. To ensure the desired outcome, one needs to optimize a range of properties of the scaffolds, including the composition, biocompatibility, biodegradation profile, and mechanical strength, as well as the porosity, morphology, and architecture. The materials suitable for tissue regeneration include natural polymers, such as collagen, gelatin, chitosan, and laminin, in addition to synthetic polymers such as polycaprolactone (PCL), poly(lactide‐*co*‐glycolide) (PLGA), polylactic acid (PLA), poly(*D*,*L*‐lactic acid) (PDLLA), and poly(*L*‐lactic acid) (PLLA), which have been approved by the U.S. Food and Drug Administration (FDA) for use in humans. In many cases, a combination of natural and synthetic polymers in the form of blend offers an effective route to the production of a ECM substitute with optimized biocompatibility, biodegradation profile, and mechanical properties.^[^ ^8^ ^]^

In combining with the stem cell therapy, a scaffold based on electrospun nanofibers should possess good biocompatibility to ensure the safety after implantation into the body for tissue regeneration. The biocompatibility of the scaffold can be improved by functionalizing the nanofibers with ECM components or bioactive molecules.^[^ ^9^ ^]^ In addition, since the scaffold is only supposed to serve as a temporary ECM substitute, it should be degraded while being replaced by the newly formed, permanent ECM. An ideal scaffold should be able to provide optimal conditions for guiding tissue reorganization while undergoing degradation within a reasonable timescale, allowing the regenerated tissue to be integrated with the host. The biodegradation profile of the scaffold is determined by not only the physical parameters of the fibers but also the surrounding environments of the implant site. The mechanical properties of the scaffold, including the strength, modulus, and stiffness, also strongly affect the behavior of stem cells interacting with it. The scaffold should also be strong enough to support the growth of stem cells while offering adequate space for the regeneration of new tissue. One can tailor the mechanical properties of the scaffold by varying the composition, diameter, and orientation of the nanofibers, as well as the bulk structure of the scaffold.^[^ ^10^, ^11^ ^]^ For example, the tensile strength of a nonwoven mat of nanofibers can be improved by welding the nanofibers at their cross points.^[^ ^12a^ ^]^ In addition, it should be emphasized that the scaffold constantly experiences compositional and structural changes in the dynamic environment of the body. As a result, the biocompatibility and mechanical properties of the scaffold will be changed, eventually altering its function and its capability to regulate the direction of the stem cell differentiation. In this regard, tracking the degradation of the scaffold in vivo and characterizing the changes in situ are of critical importance to the rational design of a scaffold.

**Figure 1:**
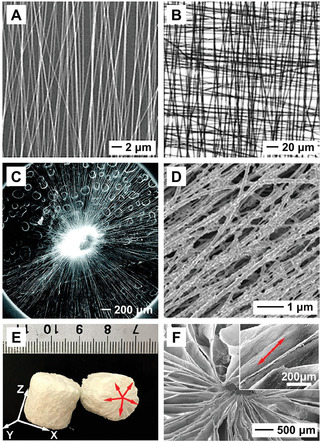
A) SEM image of PVP nanofibers collected across a gap formed between two silicon stripes. B) Optical micrograph of a grid formed by stacking two layers of uniaxially aligned PVP nanofibers, with their long axes rotated by 90°. Reproduced with permission.^[^ ^12b^ ^]^ Copyright 2003, American Chemical Society. C) SEM image of a scaffold composed of radially aligned nanofibers directly deposited on a ring collector. Reproduced with permission.^[^ ^12c^ ^]^ Copyright 2010, American Chemical Society. D) SEM images of uniaxially aligned PLGA nanofibers covered with calcium phosphate minerals. Reproduced with permission.^[^ ^13^ ^]^ Copyright 2014, American Chemical Society. E) Photograph of porous 3D cylinders derived from 2D nanofiber mats after expansion and freeze‐drying, and F) SEM image of the *X*–*Y* plane of the cylinder, which consisted of radially aligned nanofibers. Reproduced with permission.^[^ ^19^ ^]^ Copyright 2019, American Chemical Society.

The surface topography of the scaffold based on electrospun nanofibers, including the diameter, orientation, and surface roughness of the individual fibers, as well as the surface pattern of the scaffold, plays an important role in regulating the stem cell behavior and thus tissue regeneration. All these parameters also affect the biocompatibility, biodegradation profile, and mechanical properties of the scaffold. The diameter of the nanofibers can be controlled by adjusting the processing parameters during an electrospinning process and/or through post‐treatment of the nanofibers. The scaffold can also be applied to mimic the anisotropic structure of some specific types of tissues, such as tendon, nerve, and muscle, by controlling the orientation of the nanofibers, and thus the spreading and differentiation of stem cells cultured on the fibers or recruited from the tissue surrounding the defect. Uniaxially aligned nanofibers can be obtained by manipulating the mechanical force (through a rotating mandrel), electrostatic force (through a pair or array of metallic collectors), or magnetic force (through permanent magnets) during the electrospinning process.^[^ ^6^, ^12^ ^]^ For example, a uniaxially aligned array of nanofibers could be collected across a gap formed between two silicon stripes, as shown by the scanning electron microscopy (SEM) image in **Figure** **1**A for a sample made of poly(vinyl pyrrolidone) (PVP). A 3D lattice structure could be further obtained by stacking the uniaxially aligned arrays of nanofibers in a layer‐by‐layer fashion (Figure 1B). In addition, a typical scaffold comprised of radially aligned electrospun nanofibers (Figure 1C) could be fabricated by utilizing a collector containing a central point electrode and a peripheral ring electrode. The surface roughness of the individual fibers could be adjusted by manipulating the parameters during electrospinning or by post‐treatment of the nanofibers.^[^ ^6^, ^13^ ^]^ For example, uniaxially aligned PLGA nanofibers were covered with calcium phosphate minerals by immersing a plasma‐treated nonwoven mat of PLGA nanofibers in 10 × concentrated simulated body fluid, resulting in an increase in surface roughness for the individual fibers (Figure 1D).^[^ ^13^ ^]^ The surface chemistry of the electrospun nanofibers plays an important role in regulating the stem cell adhesion, growth, and differentiation. Through either physical or chemical methods, or a combination of both, various types of bioactive agents, such as ECM components, growth factors, DNA, and organic modifiers, can be adsorbed or covalently immobilized onto the surface or integrated into the bulk of nanofibers for regulating cell behavior.

**Figure 2:**
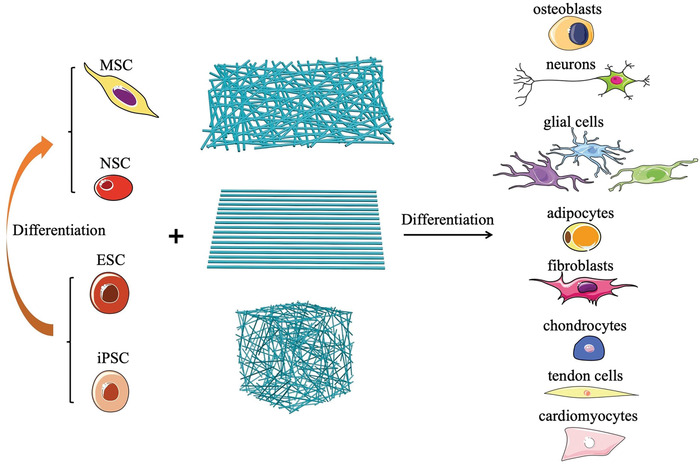
Schematics illustrating how electrospun nanofibers can be used to maneuver the differentiation of various types of stem cells toward different phenotypes.

The scaffold can be constructed with a 3D architecture to allow for the deep infiltration of stem cells, more closely mimicking the 3D structure of ECM in some tissues and providing a route to match the shape of the target tissue. Various methods have been developed to expand the 2D mat along the vertical direction for the production of a 3D sponge. One approach is to directly deposit nanofibers along the third dimension by engineering the collector for electrospinning. For example, a 3D cotton ball‐like fibrous scaffold was fabricated by collecting the nanofibers in a nonconductive spherical dish embedded with an array of metal probes.^[^ ^14^ ^]^ Another approach involves post‐treatment of the as‐spun nanofiber mat. For example, an ultralight sponge was fabricated by freeze‐drying a colloidal dispersion of short electrospun nanofibers.^[^ ^15^, ^16^, ^17^ ^]^ Jurkat cells and fibroblasts could infiltrate into the sponge and form cell clusters with the viability preserved for 30 days.^[^ ^16^ ^]^ Gas‐foaming is another promising technique for generating a 3D sponge by expanding the physically stacked mat along the third dimension.^[^ ^18^ ^]^ Different 3D architectures, including cylinders, cones, spheres, hollow spheres, and patterned structures, have all been fabricated using the gas‐foaming technique.^[^ ^19^, ^20^ ^]^ For example, Figure 1E shows a photograph of 3D porous cylinders derived from 2D nanofiber mats after expansion and freeze‐drying.^[^ ^19^ ^]^ From the SEM images in Figure 1F, the cylinder was composed of numerous thin nanofiber layers with the *X*–*Y* plane of the cylinder consisting of radially aligned nanofibers. Given the wide variety of materials that have been electrospun into nanofibers, the 3D sponges will enable the fabrication of diverse constructs for directing the differentiation of stem cells in complex 3D microenvironment. By regulating the properties of the electrospun fiber‐based scaffolding materials, one can control the migration of different types of stem cells and direct their differentiation toward various types of phenotypes (as illustrated in **Figure** **2**).

## 3 Electrospun Nanofibers for Guiding and Accelerating the Migration of Stem Cells

Cell migration plays a pivotal role in a wide variety of biological processes, including embryogenesis, wound healing, tissue renewal, and even cancer spreading.^[^ ^21^ ^]^ In the case of wound healing, it is necessary to recruit stem cells from the surrounding to the site of interest for the construction and thus regeneration of the damaged tissue.^[^ ^22^ ^]^ Cell migration is a complex process that involves multiple, sequential steps, such as adhesion, polarization, and forward movement.^[^ ^23^, ^24^ ^]^ In the case of a single cell, its migration critically depends on the establishment of polarization in terms of cytoskeletal arrangement, membrane trafficking, and signaling cascades. At the front of the cell, cytoskeletal rearrangements result in the formation of membrane protrusions, such as filopodia and lamellipodia to provide a main driving force for movement.^[^ ^24^ ^]^ The rear of the cell also actively participates in cell displacement through actomyosin contraction. The microtubule network and various components of the intracellular membrane are also organized in a polarized manner along the direction of migration. As for collective cell migration, the leader cells are polarized through interactions with the ECM and soluble factors, such as growth factors and chemokines, driving the migration.^[^ ^22^, ^24^ ^]^ The cell‐cell communication, both between the leader and follower cells and among the follower cells, further improves the efficiency of the collective movement. In general, the migration of cells, including stem cells, can be directed by various means in the form of chemical, adhesive, mechanical, and topographical cues.^[^ ^25^ ^]^

With the advent of stem cell therapy for tissue regeneration, how to regulate the migration of stem cells has gained ever increasing attention.^[^ ^5^ ^]^ To this end, electrospun nanofibers have been widely explored for directing the migration of stem cells. For example, the effect of silk fibroin fibers on the migration of mesenchymal stem cells (MSCs) was investigated.^[^ ^26^ ^]^ MSCs are stromal cells with multipotency, namely, they can differentiate into various lineages, such as chondrocytes (cartilage), myocytes (muscle), osteoblasts (bone), and adipocytes (fat). On both random and uniaxially aligned fibers, MSCs were found to migrate faster than on tissue culture plates covered with poly‐*L*‐lysine, demonstrating that electrospun fibers could indeed promote the migration of stem cells. Regardless of the fiber diameter, MSCs exhibited a higher migration efficiency on uniaxially aligned fibers relative to random fibers with the same diameter. On the aligned fibers, the cells also showed difference in terms of migration speed as the fiber diameter was varied. It was found that the aligned fibers with a diameter of 400 nm exhibited a stronger capability in promoting the migration of MSCs when benchmarked against the aligned fibers of 800 and 1200 nm in diameter.^[^ ^26^ ^]^

**Figure 3:**
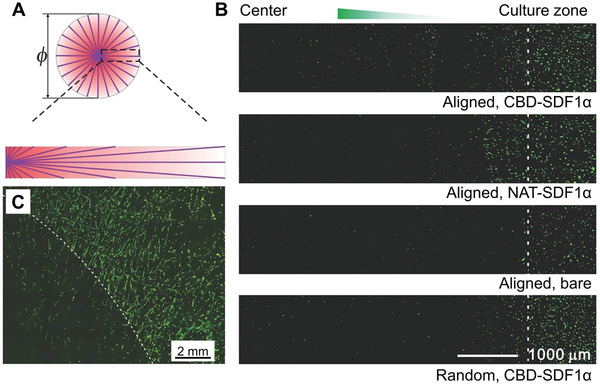
A) Schematic of a protein gradient running from the center to the periphery of a mat composed of a radially aligned array of nanofibers. B) Distributions of NSCs (stained by nestin) after culture for 1 day on the radially aligned nanofibers functionalized with CBD‐SDF1*α* or native SDF1*α* (NAT‐SDF1*α*), radially aligned nanofibers, and random nanofibers functionalized with CBD‐SDF1*α*, respectively. C) Fluorescence micrograph of NSCs cultured on the radially aligned nanofibers functionalized with CBD‐SDF1*α*. In both (B)and (C), the dashed line indicates the borderline of the seeded cells. Reproduced with permission.^[^ ^28^ ^]^ Copyright 2016, John Wiley and Sons.

The surface of a fibrous scaffold can be decorated with a bioactive factor in a graded fashion to further manipulate the migration of stem cells. In one study, a gradient in collagen‐binding domain fused with stromal cell‐derived factor‐1*α* (CBD‐SDF1*α*) was created on a nonwoven mat of random collagen nanofibers through electrohydrodynamic jet printing and then applied to guide the migration of neural stem cells (NSCs).^[^ ^27^ ^]^ When a stable and controlled gradient of the growth factor was created, a large number of NSCs were found to migrate toward the region with a greater content of CBD‐SDF1*α* on the mat. In contrast, the cells moved randomly without any preference in direction when cultured on a control sample featuring a gradient in bovine serum albumin. A gradient of SDF1*α* was also generated on radially‐aligned fibers consisting of a blend of PCL and collagen through the use of a ring collector.^[^ ^28^ ^]^ On such a scaffold, the fiber density gradually decreased from the center to periphery, enabling the creation of a gradient in density of proteins immobilized on the fibers, as illustrated in **Figure** **3**A. Upon immobilization of SDF1*α* on the collagen domains of each fiber, a radial gradient in SDF1*α* was obtained. Figure 3B compares the distributions of cells after incubation on the radially aligned fibers functionalized with CBD‐SDF1*α* or native SDF1*α*, bare radially aligned fibers, and random fibers functionalized with CBD‐SDF1*α*. On the radially aligned fibers with a gradient in CBD‐SDF1*α*, the growth factor gradient effectively accelerated the migration of NSCs from the periphery toward the center of the scaffold. In addition, the cells exhibited an elongated shape along the alignment direction of the fibers (Figure 3C). A similar effect was also observed in promoting the directional migration of human MSCs (hMSCs) on a radially aligned fibrous scaffold coated with polydopamine.^[^ ^29^ ^]^ To achieve the desirable migration behavior for stem cells, the pattern of the gradient and the amount and type of the growth factors still need to be systemically investigated and further optimized.

These studies offered valuable information about the design and rational fabrication of scaffolds with optimal performance with regard to the migration and self‐organization of stem cells. Unfortunately, the extent of cell recruitment is rather low in most experimental and clinical studies. To improve the efficacy of stem cell therapy, elucidation of the mechanisms that govern the migration of stem cells to the injured areas of interest is essential for the establishment of new clinically acceptable therapeutic paradigms. In addition, high matrix density and stiffness of adult dense connective tissues can restrict the mobility of endogenous stem cells, impeding tissue regeneration after injury. In this case, some enzymes that can digest or break the dense barriers should be loaded and then released from the scaffolds to help improve the cell migration.

## 4 Electrospun Nanofibers for Directing the Differentiation of Stem Cells

Electrospun nanofibers are well‐suited for directing the differentiation of stem cells as these cells are responsive to the topographic features presented by the surrounding ECM and can change their phenotypes according to the microenvironment. Electrospun nanofibers can be engineered to imitate the features of the stem cell niches for controlling the fate of stem cells.^[^ ^30^, ^31^, ^32^ ^]^ In general, proteins in the ECM, such as collagen, laminin, and fibronectin, assemble in their hydrated environment into 3D fibrillar architectures, which can be reproduced through the use of electrospun nanofibers. Electrospun nanofibers can also be incorporated with biochemical cues, such as growth factors, surface modifiers,^[^ ^33^, ^34^, ^35^ ^]^ and adhesive signals to control the spatial distribution of focal adhesions for the cells. MicroRNA or DNA agents can also be integrated with electrospun nanofibers for gene silencing or delivery.^[^ ^36^, ^37^ ^]^ The biochemical cues can be recognized by cell surface receptors through integrin conformational changes and clustering, activating the focal adhesion kinase (FAK) recruitment and phosphorylation and ultimately regulating differentiation pathways. A recent study has highlighted the upregulation of FAK expression for stem cells after incubation on aligned nanofibers and the decrease of cell elongation caused by the FAK silencing through small hairpin RNA.^[^ ^38^ ^]^ The biochemical and mechanotransduction mechanisms associated with the interaction between stem cells and nanofibers are numerous, involving a variety of microenvironmental factors, such as material stiffness,^[^ ^39^, ^40^ ^]^ cues mediated by integrin domains, contractility of the cytoskeleton, and nuclear deformations.^[^ ^41^, ^42^, ^43^, ^44^, ^45^ ^]^ In addition, scaffolds made of electrospun nanofibers are large and thick enough to show sufficient mechanical robustness for convenient manageability and for providing cells and tissues with structural support. In the following, main findings related to instructive scaffolds made by electrospun nanofibers and tested with stem cells are summarized, with classification in terms of the specific type of stem cells used in the experiments.

### 4.1 MSCs

MSCs exist in many types of tissues, including bone marrow, adipose, and synovium, and they can differentiate into almost any end‐stage lineage cells when seeded on/in a proper scaffold.^[^ ^46^ ^]^ The effect of electrospun nanofibers on the fate of MSCs has been investigated in a number of studies.^[^ ^47^, ^48^, ^49^, ^50^, ^51^, ^52^, ^53^, ^54^, ^55^, ^56^, ^57^, ^58^, ^59^, ^60^ ^]^ MSCs have multiple differentiation potential toward osteogenic, chondrogenic, or adipogenic lineages, as well as cells with an oligodendrocyte or motor neuron‐like phenotype, for the healing of various tissues such as bone, cartilage, adipose, muscle, nerve, and myocardium.^[^ ^48^, ^49^, ^61^ ^]^

**Figure 4:**
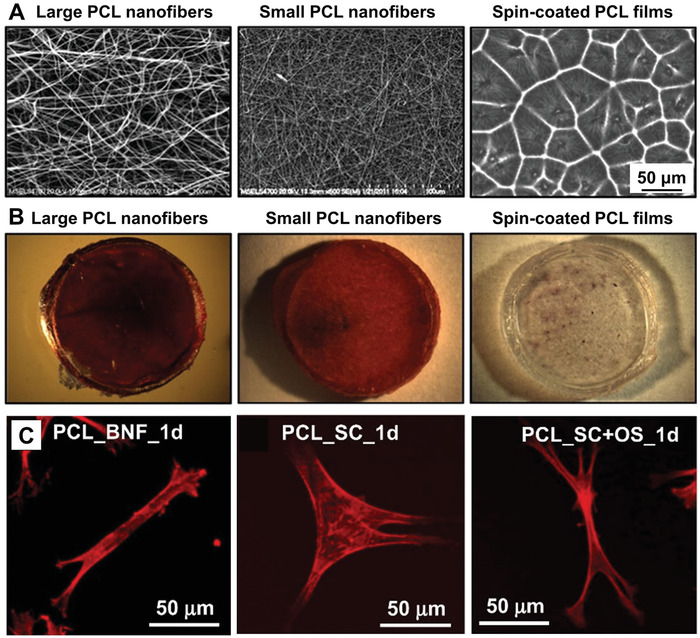
A) Micrographs of different types of scaffolds in the form of electrospun PCL nanofibers with a large (910 nm) or small (326 nm) diameter and spin‐coated film, respectively. Fibers are imaged by SEM, while the spin‐coated film is imaged by phase‐contrast microscopy. B) Stereomicrographs of Ca^2+^ staining, with the use of Alizarin red, for the osteogenesis of bone‐derived hMSCs on the different types of scaffolds after 50 days of culture in the absence of osteogenic supplements. C) Fluorescence micrographs of hMSCs after 1 day of culture on the PCL nanofibers with a diameter of 910 nm (PCL_BNF_1d) and on the spin‐coated film in the absence (PCL_SC_1d) and presence of osteogenic supplements (PCL_SC+OS_1d), respectively. Reproduced with permission.^[^ ^57^ ^]^ Copyright 2011, Elsevier.

The nanoscale features of the electrospun fibers play an important role in regulating the fate of MSCs. Various types of electrospun nanofibers made of PCL,^[^ ^47^ ^]^ collagen,^[^ ^53^ ^]^ gelatin,^[^ ^54^ ^]^ and graphene oxide‐doped PLGA^[^ ^55^ ^]^ have been proved to be able to enhance the expression of osteogenic markers in MSCs relative to tissue culture plates or casting films. Some of the scaffolds have been used as bone grafts in a rat model and as cartilage grafts in a swine model, respectively.^[^ ^56^ ^]^ In one study, the behaviors of bone‐derived hMSCs on scaffolds with different microscopic structures were compared, as shown in **Figure** **4**.^[^ ^57^ ^]^ Osteogenesis of the hMSCs was observed on scaffolds made of electrospun nanofibers after 50 days of incubation in the absence of osteogenic supplements. However, there was no indication of osteogenic differentiation for the hMSCs when incubated on scaffolds fabricated using other methods, including salt‐leaching, gas foaming, thermally induced phase‐separation combined with gas foaming, and spin coating. In addition, the morphology of the scaffold was found to prevail over the surface composition in determining the cell fate in a certain content, with nanofibrous systems being uniquely able to drive osteogenic commitment in the absence of osteogenic supplements. The actin and microtubule filaments are essential for the differentiation of MSCs into osteoblasts on the nanofibers.^[^ ^58^ ^]^ Osteogenic differentiation was also observed when adipose‐derived MSCs were incubated on scaffolds made of mineralized PCL‐gelatin core–sheath fibers.^[^ ^59^ ^]^ In another study, adipose‐derived hMSCs incubated on electrospun PCL nanofibers showed increased cellular lipid accumulation and higher content of hormone sensitive lipase relative to the group on tissue culture plates, indicating the potential of establishing an adipocyte cell model through the use of electrospun nanofibers.^[^ ^60^ ^]^ Instructive scaffolds for controlling the differentiation of MSCs can be realized by adjusting the composition of the fiber matrix, such as by doping nanofibers with adhesive molecules or with conductive polymers like polypyrrole and polystyrenesulfonate to endow the scaffold with electrical activity.^[^ ^62^ ^]^

The orientation of the nanofibers also affects the differentiation of the MSCs. In one study, adipose‐derived MSCs were encouraged for tenogenic differentiation after incubation on uniaxially aligned nanofibers relative to random nanofibers.^[^ ^63^ ^]^ However, the orthogonally oriented nanofibers promoted the osteogenic differentiation of bone‐derived MSCs.^[^ ^64^ ^]^ In addition to straight nanofibers, coiled nanofibers were found to promote the differentiation of bone‐derived MSCs toward myofiblasts.^[^ ^65^ ^]^

**Figure 5:**
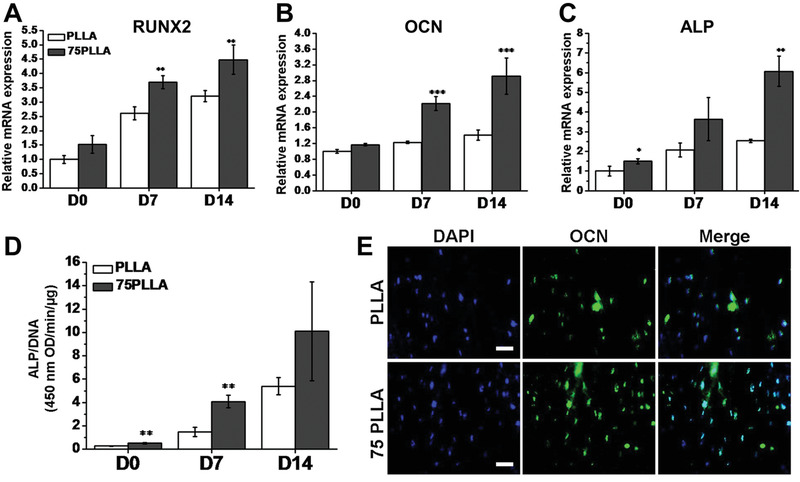
Osteogenic differentiation of hMSCs cultured on PLLA nanofibers without and with annealing at 75 °C (75PLLA), respectively. The cells were cultured in basal medium for 7 days and then induced in an osteogenic differentiation medium for additional 14 days. A–C) Expression levels of mRNA transcripts of bone‐associated markers in hMSCs cultured on the two different types of scaffolds. D) ALP activity in hMSCs measured and normalized by DNA content. E) Immunofluorescence labeling of osteocalcin (OCN, green) in hMSCs with cell nuclei stained with 4′,6‐diamidino‐2‐phenylindole (DAPI, blue). Scale bar = 50 µm. ^∗^ *p* < 0.05, ^∗∗^ *p* < 0.01, ^∗∗∗^ *p* < 0.001, *n* = 3. Reproduced with permission.^[^ ^40^ ^]^ Copyright 2016, Elsevier.

The mechanical properties of the scaffolds based on electrospun nanofibers also show great influence on the differentiation of MSCs.^[^ ^66^, ^67^ ^]^ Scaffold with an elastic modulus of brain (0.1–1 kPa), muscle (∼8–17 kPa), or stiff cross‐linked collagen (25–40 kPa) is inclined to direct the differentiation of MSCs toward neural, myoblast, or osteoblast‐like lineages, respectively.^[^ ^66^ ^]^ Stiff substrates direct the cells toward the osteogenic lineage, while soft ones direct the cells toward the tenogenic lineage. In one study, the Young's modulus of a mat made of electrospun PLLA nanofibers was increased from 77.4 ± 17.7 to 1124 ± 119 MPa by annealing at 75 °C without changing the material chemistry.^[^ ^40^ ^]^ After osteogenic induction for 7 and 14 days, the MSCs incubated on the annealed nanofibers showed an improved osteogenic differentiation relative to those incubated on the pristine nanofibers, as proven by the upregulation of relative mRNA levels of bone‐related markers expressed by cells (**Figure** **5**A–C). Higher alkaline phosphatase (ALP) activity was detected from the cells incubated on annealed nanofibers (Figure 5D). In addition, a greater intensity of osteocalcin labeling was observed in the cells incubated on the annealed nanofibers (Figure 5E). The increased stiffness favorably induced the differentiation of MSCs into the osteogenic lineage through the macrophage migration inhibitory factor (MIF)‐mediated AKT/yes‐associated protein (YAP)/runt‐related transcription factor 2 (RUNX2) pathway. Cells on stiffer substrates increasingly activated AKT and YAP and upregulated transcript expression of YAP target genes. Furthermore, MIF was increasingly produced by the cells on stiffer substrates.

The surface of electrospun nanofibers can be functionalized with functional groups or bioactive agents to interact with MSCs for directing their growth and differentiation. In one study, the surfaces of PLGA fibers were functionalized with bone‐forming peptide 1 derived from the immature region of bone morphogenetic protein 7 to enhance alkaline phosphatase activity and calcium deposition for the cultured hMSCs.^[^ ^68^ ^]^ After patching mouse calvarial defects with the fibers, bone formation was significantly improved. Different types of factors can be coimmobilized on the surface of electrospun fibers to give a synergetic effect in controlling the differentiation of MSCs. For instance, endogenous bone morphogenetic protein‐2 and vascular endothelial growth factor were deposited on a nanofiber membrane to promote sustained spatial angiogenesis and osteogenesis of bone‐derived hMSCs when cultured in basal medium while presenting a higher angiogenic effect in vivo.^[^ ^69^ ^]^ A layer‐by‐layer technique was applied to graft different types of peptides into discrete layers of electrospun fibers for controlling cell adhesion and differentiation sequentially.^[^ ^70^ ^]^ Gene therapy also has a significant potential to deliver biological agents as specific signals for controlled MSC differentiation. RUNX2 is a central gene involved in the osteoblast phenotype induction. RUNX2‐loaded liposomes were immobilized on the surface of electrospun PCL nanofibers to induce a long‐term gene expression of RUNX2 by the cultured bone‐derived hMSCs and an enhanced level of metabolic activity and protein synthesis.^[^ ^71^ ^]^ Furthermore, osteogenic differentiation of bone‐derived hMSCs was also achieved by the overexpression of other osteogenic markers in a medium free of osteogenic supplementation.

To further improve the functionality of the scaffolds, various types of growth factors and/or bioactive nanoparticles have been incorporated into the nanofibers. For example, doping silk fibroin nanofibers with bone morphogenic protein‐2 and hydroxyapatite could promote the osteogenic differentiation of MSCs, leading to higher transcript levels of bone morphogenic protein‐2, bone sialoprotein, and collagen I, all of which are bone‐specific markers.^[^ ^51^ ^]^ Even in the absence of exogenous osteogenic supplements, electrospun nanofibers doped with hydroxyapatite and/or tricalcium phosphate were found to promote osteogenic differentiation of MSCs.^[^ ^72^, ^73^, ^74^ ^]^ MSCs could also be differentiated into neuronal lineages expressing neuronal proteins after incubation on PCL^[^ ^75^, ^76^ ^]^ and on PLLA‐*co*‐PCL/collagen^[^ ^77^ ^]^ nanofibers, both of which were loaded with retinoic acid. In another study, vascular endothelial growth factor was encapsulated in gelatin particles and then integrated with PCL nanofibers.^[^ ^78^ ^]^ The as‐fabricated scaffold could direct the differentiation of MSCs toward endothelial cells with tubular morphology. Electrospun nanofibers can also serve as a carrier of genes for transfecting MSCs, maneuvering the directional differentiation.^[^ ^79^, ^80^ ^]^ For example, the sustained release of siRNA targeting RE‐1 silencing transcription factor, a master negative regulator of neurogenesis, from PCL nanofibers promoted the neuronal differentiation of hMSCs.^[^ ^81^ ^]^

The cellular constructs consisting of MSCs or differentiated MSCs and scaffolds made of electrospun nanofibers have been applied to a variety of in vivo studies for tissue regeneration. For example, MSCs were incubated on electrospun PLLA fibers to construct a cellular graft for vascular tissue regeneration.^[^ ^82^ ^]^ After the cellular graft had been implanted into the damaged common carotid artery of rats, well‐organized endothelial and smooth muscle layers were observed, resembling the structure of arteries. In another study, MSCs were incubated on electrospun PLLA nanofibers and then implanted into a surgical cavity after glioblastoma resection in a mouse model.^[^ ^83^ ^]^ By this approach, a better retention of MSCs in the surgical cavity was achieved, leading to a better delivery of antitumor proteins and ultimately a reduction of the regrowth of residual glioblastoma foci.

MSCs can also be derived from iPSCs and then seeded onto nanofibers to perform further transdifferentiation and/or implantation. In one study, iPSCs were first induced into MSCs on a smooth plastic surface, and the obtained cells were then differentiated into osteoblasts on nanofibers made of a blend of collagen, chitosan, and hydroxyapatite.^[^ ^84^ ^]^ The cellular scaffolds effectively promoted bone regeneration in cranial defects of mice. In another study, MSCs derived from iPSCs were incubated on uniaxially aligned nanofibers and then differentiated into tenocyte‐like cells.^[^ ^85^ ^]^ The cellular scaffolds significantly improved the structural and mechanical properties of the repaired tendon in a rat model for Achilles tendon repair.

Although many studies have been reported, the safety of MSCs‐related therapies remains a major concern for clinical applications due to the potential risks of MSCs, such as tumorigenicity, proinflammation, and fibrosis. In the dynamically changing environment of a body, the phenotypes of MSCs or the derived cells may change as well. For example, MSCs can also be differentiated into myofibroblasts, producing fibrotic reactions instead of tissue repair. In order to improve the therapeutic effects of MSCs and reduce the potential risks, it is essential to precisely control the cultural environment of MSCs and select the appropriate combination of scaffold and induction factors for achieving accurate administration.

### 4.2 Neural Stem Cells

**Figure 6:**
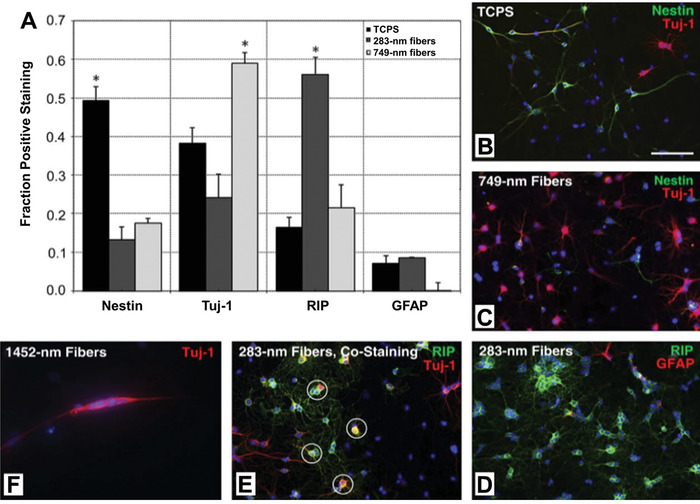
Immunofluorescence analysis of rat NSCs cultured on tissue culture plates (TCPS), electrospun nanofibers with an average diameter of 283 (283 nm fibers) and 749 nm (749 nm fibers), respectively. A) The fraction of cells positively staining with nestin (neural stem/progenitor cell marker), Tuj‐1 (neuronal marker), RIP (oligodendrocyte marker), and GFAP (astrocyte marker), respectively, after culture on the different types of substrates. ^∗^ *p* < 0.05 versus all other samples. B−D) The corresponding micrographs for each substrate. E) The micrograph of cells costained with RIP (green) and Tuj‐1 (red), highlighted by circles, on the 283 nm fibers. F) The micrograph of cells cultured on 1452 nm fibers, which were statistically unquantifiable due to the low viability of cells. Cell nuclei are stained by DAPI (blue) in all the micrographs. The scale bar in (B) is 100 µm and applies to all the micrographs. Reproduced with permission.^[^ ^90^ ^]^ Copyright 2009, Elsevier.

NSCs can self‐renew and differentiate along different neural lineages, including neurons, astrocytes, and oligodendrocytes.^[^ ^86^, ^87^, ^88^, ^89^ ^]^ When subjected to appropriate biochemical and topographical cues, the NSCs or NSC‐derived cells can be transplanted into damaged regions with scaffolds made of electrospun nanofibers for the repair of brain, spinal cord, and peripheral nerve injuries. The fiber diameter, in particular, is found to play a critical role in determining the proliferation and lineage specification of NSCs. In one study, rat hippocampus‐derived adult NSCs were seeded on laminin‐coated polyethersulfone fibers with different diameters.^[^ ^90^ ^]^ In the differentiation medium containing 1 × 10^−6^ m retinoic acid and 1% fetal bovine serum, the NSCs assumed glial cell shape and preferentially differentiated into oligodendrocytes on fibers with a diameter of ≈280 nm, whereas they elongated on 749 and 1452 nm fibers and preferentially differentiated into the neuronal lineage. As shown in **Figure** **6**, the oligodendrocyte differentiation of rat NSCs increased by 40% on fibers with a diameter of ≈280 nm and the neuronal differentiation increased by 20% on fibers with a diameter of ≈749 nm when benchmarked against the control cultured on a tissue culture plate.

The topography of the electrospun nanofibers also affects the behavior of NSCs as they show different responses when incubated on the random and aligned nanofibers, respectively. At average diameters of 600 and 1600 nm, aligned polysulfone fibers were more effective than random fibers in promoting the differentiation of NSCs derived from human embryonic stem cells (ESCs) toward the Schwann cell lineage.^[^ ^91^ ^]^ Nerve conduits seeded with NSCs derived from iPSCs resulted in accelerated repair of sciatic nerve injuries in rats.^[^ ^92^ ^]^ PCL/gelatin fibers were found to direct and promote neurite outgrowth from neonatal mouse cerebellum C17.2 stem cells.^[^ ^93^ ^]^ When cultured on PLLA nanofibers, NSCs showed a rich phenomenology, and the rate of neuronal differentiation increased with the reduction in fiber diameter.^[^ ^94^ ^]^ The viability and proliferation of C17.2 stem cells were found to be optimal on fibers with diameters of ≈500 and 1100 nm, respectively, when the fibers were uniaxially aligned and random,^[^ ^95^ ^]^ highlighting a complex interplay of dimensional and anisotropic topographical cues in affecting cellular behavior.

**Figure 7:**
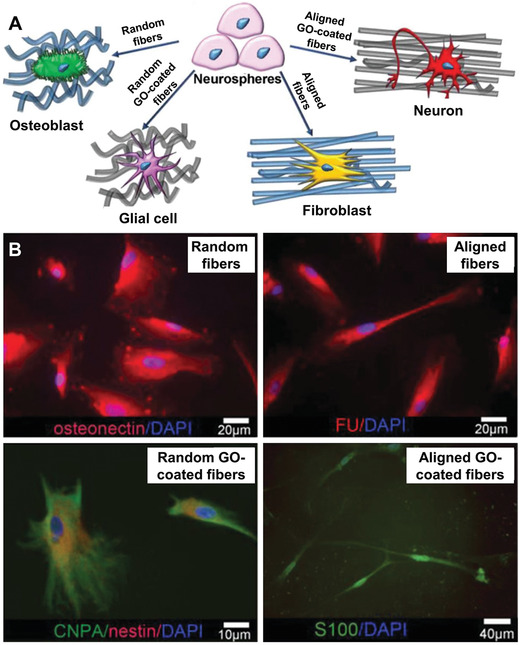
A) Schematic illustrations of the differentiation of primary neurospheres directed by different types of fibrous scaffolds with or without graphene oxide (GO) functionalization in a random or aligned array, respectively. The scaffolds could drive the differentiation toward glial cells and neurons, or revert neuronal precommitment, inducing fibroblasts and osteoblasts differentiation. B) The corresponding micrographs after neurospheres are cultured on random fibers, aligned fibers, random fibers coated with GO, and aligned fibers coated with GO, respectively, with staining against osteonectin (red), fibroblast surface protein (FU) (red), CNPase (CNPA) (green)/nestin (red), and the neuronal marker S100 (green). Cell nuclei are stained by DAPI (blue) in all micrographs. Reproduced with permission.^[^ ^97^ ^]^ Copyright 2018, John Wiley and Sons.

Other types of neural‐related cells with differentiation capabilities also deserve to be mentioned in this framework. In one study, retinal progenitor cells were shown to preferentially differentiate into retinal neurons, including photoreceptors, when cultured on nanofibers made of a blend of silk fibroin and PLA‐*co*‐PCL.^[^ ^96^ ^]^ In another study, neurospheres derived from dental pulp stem cells were demonstrated with different differentiation propensity when cultured on nanofibers featuring different alignments and further functionalized with graphene oxide.^[^ ^97^ ^]^ As shown in **Figure** **7**, the instructive cues provided by the surface could lead to the different lineage commitments, including reprogramming features toward osteoblasts, glial cells, fibroblasts, and neurons even in basal medium conditions, with the expressions of osteonectin (bone morphogenetic protein), CNPase (glial commitment), fibroblast surface protein, and neuronal marker S100, respectively. These studies have demonstrated the possibility of using electrospun nanofibers to regulate the growth and differentiation of NSCs. However, more detailed mechanistic studies are still required to explain how these different types of cues modulate the signaling pathways and cascades that are primarily involved in the differentiation process of NSCs.

### 4.3 Embryonic Stem Cells

ESCs are pluripotent and can differentiate into any of the three germ layers. However, it still remains a grand challenge to achieve a tight control over their differentiation into lineages for long‐term viable tissues. Human ESCs have been seeded onto PCL,^[^ ^98^ ^]^ PCL/gelatin, PCL/collagen,^[^ ^99^ ^]^ PLLA, collagen‐grafted polyethersulfone,^[^ ^100^ ^]^ and PLGA^[^ ^101^ ^]^ nanofibers, which could effectively support their expansion, possibly with significant retention of stemness features. In one study, mouse ESCs cultured on nanofibers showed enhancement in proliferation and self‐renewal relative to tissue culture plates, which was correlated with the small GTPase Rac activation, the phosphoinositide 3‐kinase pathway activation, and the expression of Nanog homeoprotein required for maintenance of pluripotency.^[^ ^102^ ^]^ Surface chemistry is another major tool for modulating the formation and self‐renewal of ESC colonies. Understanding how surface properties can be combined with biological factors to direct cellular fate can lead to methods for improving the retention of pluripotency and generating a large number of high‐quality ESCs. For example, ESCs could respond to the surface functional groups of an octadiene‐allylamine plasma polymer gradient platform.^[^ ^103^ ^]^ Expressions of the stem cell markers alkaline phosphatase and Oct4 were more strongly retained on the octadiene plasma polymer‐rich regions of the gradient. More compact, multilayered colonies arose along the gradient with the decrease in allylamine content. It is still challenging, however, to identify the optimal surface chemistry of electrospun fibers for ESC maintenance.

**Figure 8:**
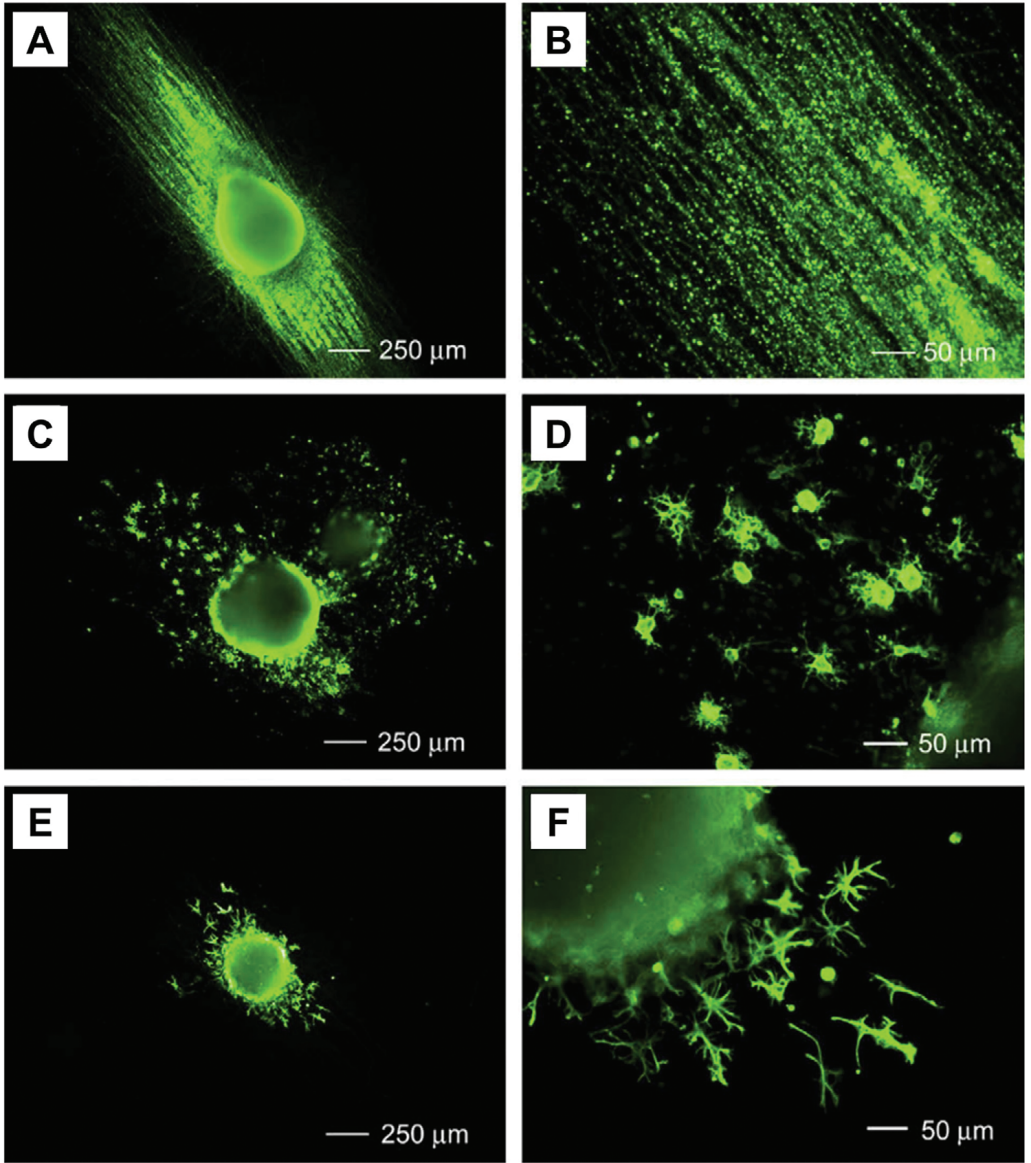
Immunofluorescence micrographs of embryoid bodies after culture for 14 days on aligned PCL nanofibers. Staining is performed for mature cell markers including A,B) Tuj‐1 (neuronal marker), C,D) O4 (oligodendrocyte marker), and E,F) glial fibrillary acidic protein (GFAP, astrocyte marker), respectively. Reproduced with permission.^[^ ^106^ ^]^ Copyright 2009, Elsevier.

By controlling the surface properties and culture media, electrospun nanofibers have been shown to enhance the differentiation of ESCs into various types of cell lineages relative to flat surfaces, including osteoblasts, cardiomyocytes,^[^ ^104^ ^]^ adipocytes,^[^ ^105^ ^]^ and neurons.^[^ ^106^ ^]^ For example, ESCs could interact strongly with PLLA nanofibers, as evident from the protrusions formed by cells, together with osteogenic differentiation promoted by osteoinductive supplements.^[^ ^107^ ^]^ In another study, electrospun nanofibers could enhance the differentiation of mouse ESCs into neuronal lineages and promote neurite outgrowth.^[^ ^106^ ^]^ Several specific neural lineages, including neurons, oligodendrocytes, and astrocytes, were obtained after differentiation, as shown in Figure 8.^[^ ^106^ ^]^ Aligned nanofibers turn out to be an especially interesting platform for neural differentiation, in view of developing an approach based on stem cells for the repair of nerve injuries. In addition, the aligned morphology could increase the neuronal differentiation. Mouse ESCs cultured on aligned PLGA fibers had statistically higher nestin expression than that on random fibers.^[^ ^108^ ^]^ The surface of electrospun nanofibers can be functionalized with specific bioactive molecules to further manipulate the differentiation of ESCs. In one study, the surfaces of uniaxially aligned PLLA fibers were functionalized with Tyr‐Ile‐Gly‐Ser‐Arg (YIGSR) peptide. When used to the incubation of mouse ESCs, the fraction of cells expressing neuron‐specific markers and the level of neurite extension were both significantly increased relative to that on random, bare fibers.^[^ ^109^ ^]^ Similarly, functionalization of PCL fibers with Gly‐Tyr‐Ile‐Gly‐Ser‐Arg (GYIGSR) peptide increased the rate of neuronal differentiation of mouse ESCs relative to laminin‐coated fibers.^[^ ^110^, ^111^ ^]^ In another study, glycosaminoglycans (GAGs), heparan sulfate, was ionically immobilized onto the surface of PLGA fibers. The bound GAGs retained the ability to interact with GAG‐binding molecules and presented GAG sulfation motifs, promoting the extensive neural differentiation of mouse ESCs in comparison with unfunctionalized fibers.^[^ ^112^ ^]^

### 4.4 Induced Pluripotent Stem Cells

iPSCs represent another type of pluripotent stem cell with an ESC‐like state, which are usually derived by genetically reprogramming adult cells with transcription factors. For this reason, they provide an excellent cell source for personalized regenerative medicine. At the same time, it is crucial to minimize the safety concerns of iPSCs in vivo (e.g., tumorigenesis and teratoma formation) by precisely modulating their behaviors. Various attempts have been made to adjust the properties of electrospun nanofibers for either keeping the iPSCs at the undifferentiated states or directing them to differentiate into specific lineages.^[^ ^113^, ^114^, ^115^ ^]^ For example, compared with tissue culture plates, gelatin‐coated electrospun PCL nanofibers promoted the differentiation of murine iPSCs into cardiomyocytes.^[^ ^116^ ^]^

The surface topography of electrospun nanofibers, including their orientation and pattern, greatly affect the fate of iPSCs. For example, the differentiation of iPSCs toward cardiac cells was promoted in the case of aligned PLGA nanofibers relative to random nanofibers.^[^ ^117^ ^]^ The cardiac differentiation of iPSCs was also achieved using a honeycomb‐compartmented monolayer of gelatin nanofibers.^[^ ^118^ ^]^ Biochemical cues were also integrated with electrospun nanofibers to direct the differentiation of iPSCs. In one study, human iPSCs were induced to perform neural differentiation when incubated on PCL fibers functionalized with retinoic acid.^[^ ^114^ ^]^ Electrochemical cue represents another effective tool for regulating the differentiation of iPSCs, which can be combined with the manipulation of fiber topography to achieve the optimal outcome. By applying electrical signals to uniaxially aligned fibers made of polyaniline and polyetersulfone, iPSCs incubated on the fibers were induced to differentiate into cTnT^+^ cells with increased expression of cardiac‐related transcription factors relative to the group involving random nanofibers.^[^ ^119^ ^]^

**Figure 9:**
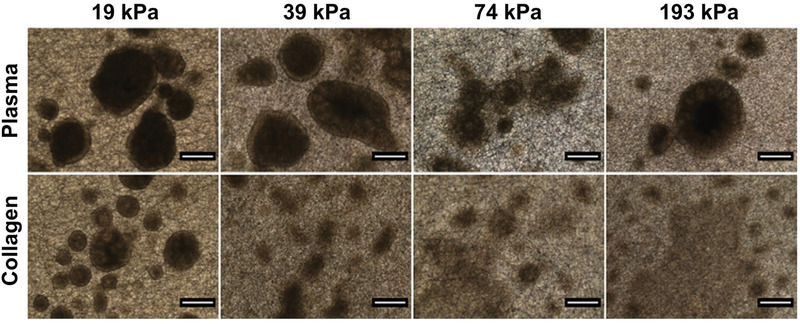
Optical micrographs of colonies of human iPSCs after culture on electrospun nanofibers with variable Young's moduli and surface functionalization. The nanofibers were either treated with plasma (top row, where the colonies are relatively small and clearly defined in terms of shape) or conjugated with collagen (bottom row, where the colonies are large and more spread‐out). Scale bar = 500 µm. Reproduced with permission.^[^ ^39^ ^]^ Copyright 2015, Elsevier.

The formation and morphology of colonies from the iPSCs are also affected by the properties of electrospun nanofibers, with the sphericity of the colony, in turn, affecting the spontaneous differentiation.^[^ ^39^, ^120^ ^]^ In particular, the mechanical properties and the surface functionalization of the electrospun fibers show significant influence on the morphology of the colonies generated from iPSCs. As shown in **Figure** **9**, when the cells were cultured on plasma‐treated electrospun fibers for 9 days, the obtained colonies were relatively smaller and clearly defined in their shape in comparison to larger and more spread colonies obtained on collagen‐conjugated fibers.^[^ ^39^ ^]^ The Young's modulus of the fibers significantly affected the shape and the size of the colonies. Well‐defined, 3D colonies could be obtained on the electrospun fibers with suitable surface modulus. In another study, neural induction of the iPSCs was initially enhanced when incubated on soft substrates.^[^ ^121^ ^]^ As the differentiation progressed, the stiffer substrates promoted the differentiation toward neural progenitor cells and motor neurons. The dynamic changes in the stiffness of the fibrous scaffold may provide a route for enhancing the differentiation efficiency toward specific lineage. The iPSCs can also be induced into other types of progenitor cells, for example, neural progenitor cells, which can further undergo neural transdifferentiation on the electrospun nanofibers.^[^ ^122^ ^]^

**Figure 10:**
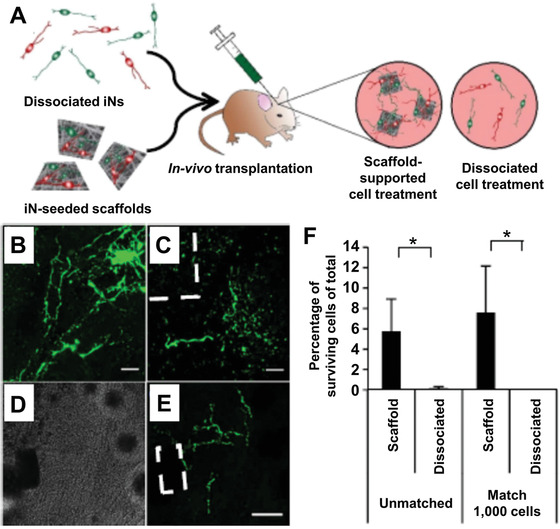
The in vivo survival of induced neuronal cells (iNs) supported by scaffolds based on electrospun nanofibers. A) A schematic indicating that dissociated iNs and a iNs‐seeded scaffold were injected and implanted into the mouse striatum, respectively. B−E) Micrographs of the green fluorescent protein (GFP)‐expressing surviving cells at 3 weeks post‐transplantation. Cells were found for both groups of B) injecting dissociated cells and C) implanting with the iN‐seeded scaffolds. The scaffolds were highlighted by dashed lines in (C) and (E). The scale bars are 25 µm in (B) and (C) and 100 µm in (E), respectively. F) The quantification of surviving cells. The “matched” subpanel compares survival percentage on the base of a similar magnitude of initially injected cells (1000). ^∗^ *p *< 0.05. Reproduced under the terms of the Creative Commons CC BY license.^[^ ^123^ ^]^ Copyright 2016, Springer Nature, Published by Nature Publishing Group.

In vivo applications of iPSCs toward tissue engineering and regenerative medicine are rapidly becoming a reality. In one study, iPSCs were differentiated into neuronal cells on electrospun nanofibers through ectopic expression of NeuroD1 transcription factors.^[^ ^123^ ^]^ The induced neural cells supported on the fibers were then transplanted into mouse striatum, resulting in the enhancement for the neuronal delivery and the action potential firing relative to the group of injecting dissociated cells (**Figure** **10**). A higher fraction of survival cells was found with the use of the supporting scaffolds.

Keeping faith with their peculiar advantage of combining topographical and chemical cues with robustness and structural support for handling bioconstructs, electrospun nanofibers are now entering a new stage for controlling the behaviors of ESCs and iPSCs, which will be entirely developed in vivo and more and more closely approach the clinic.

## 5 Conclusions and Outlook

Owing to their unique characteristics, including ECM‐mimicking structure, large surface area, and high porosity, electrospun nanofibers have been widely applied as functional scaffolds to control the behavior of stem cells for tissue engineering and regenerative medicine. By engineering the surface chemistry, topography, and architecture of the scaffolds, as well as the incorporation of other biochemical and electrochemical cues, various types of scaffolds have been designed to maneuver the migration and differentiation of various types of stem cells.

Although electrospun nanofibers offer a significant aid to the application of stem cells in tissue regeneration, challenges still exit. One issue is the difficulty of producing large amounts of uniform nanofibers to ensure reproducibility for the obtained scaffolds and thus an accurate control of the stem cell behavior. The reproducible manufacturing of high‐quality scaffolds based on electrospun nanofibers critically depends on both the accuracy and reproducibility of a production process. It has also been difficult to produce nanofibers with diameter below 50 nm, which may be desirable to better mimic the structure of some specific ECM components. Another challenge is the construction of scaffolds with well‐defined 3D architecture over large volumes, such as ordered structure and controllable pore size, which are important to improve the infiltration of stem cells and ultimately promote the integration of repaired tissues with the host. In addition, an optimal combination of the scaffold with the suitable biochemical and electrochemical cues still needs to be further investigated for precisely controlling the migration and directional differentiation of stem cells. It is desirable to regulate the fate of cells without the use of induction media during in vitro incubation. In this case, one should try to combine the optimized nanofibers together with the controlled release of soluble factors from the scaffolds in a well‐defined sequence to mimic the realistic environment in the native tissue.

The application of electrospun fibers for in vivo stem cell delivery can be complex since a surgical process is usually required for their implantation into the target sites. One potential strategy to overcome this limitation is to develop injectable fiber‐based scaffolds, which have attracted some attention in recent years. By either cryo‐cutting or homogenization, aqueous dispersions of short segments of electrospun fibers can be obtained, which can be directly injected and printed out,^[^ ^124^ ^]^ or electrosprayed into liquid nitrogen to form injectable fibrous microspheres.^[^ ^125^ ^]^ Another promising method is to produce sliding fibers by coating the electrospun, full‐length fibers with a layer of lubricating hydrogel material, endowing the fibers with gel‐like property, and it has been demonstrated to deliver NSCs.^[^ ^126^ ^]^ These injectable fibers can replace the implantation surgery with a minimally invasive injection method and can form any desired shape to match irregular defects. Further studies should be conducted to investigate the efficacy of these systems in supporting the differentiation of stem cells at their local biochemical and biomechanical environment after injection into the target site.

Finally, in addition to the safety concern arising from the scaffold, the safety of stem cells in vivo also needs to be determined using larger controlled clinical trials as the development of cancer caused by the stem cells is a continuous process. Addressing these issues will lead to a novel class of optimized electrospun nanofibrous scaffolds, providing new opportunities for pushing these systems toward clinical applications involving stem cell therapies and new areas in the broad field of biomedicine.^[^ ^127^ ^]^

## Conflict of Interest

The authors declare no conflict of interest.
